# Universal Quake Statistics: From Compressed Nanocrystals to Earthquakes

**DOI:** 10.1038/srep16493

**Published:** 2015-11-17

**Authors:** Jonathan T. Uhl, Shivesh Pathak, Danijel Schorlemmer, Xin Liu, Ryan Swindeman, Braden A. W. Brinkman, Michael LeBlanc, Georgios Tsekenis, Nir Friedman, Robert Behringer, Dmitry Denisov, Peter Schall, Xiaojun Gu, Wendelin J. Wright, Todd Hufnagel, Andrew Jennings, Julia R. Greer, P. K. Liaw, Thorsten Becker, Georg Dresen, Karin A. Dahmen

**Affiliations:** 1 Retired, Los Angeles, CA,; 2 Department of Physics, University of Illinois at Urbana Champaign, 1110 West Green Street, Urbana, IL 61801; 3 German Research Centre for Geosciences, Telegrafenberg, 14473 Potsdam, Germany; 4 Department of Earth Sciences, University of Southern California, Los Angeles, CA 90089; 5 Department of Physics and Center for Nonlinear and Complex Systems, Duke University, Durham, North Carolina, 27708-0305 USA; 6 Department of Physics, University of Amsterdam, 1090 GL Amsterdam, Netherlands; 7 Department of Mechanical Engineering, One Dent Drive, Bucknell University, Lewisburg, PA 17837; 8 Department of Chemical Engineering, One Dent Drive, Bucknell University, Lewisburg, PA 17837; 9 Department of Materials Science and Engineering, Johns Hopkins University, 3400 North Charles Street, Baltimore, Maryland 21218; 10 Department of Materials Science, Caltech, MC 309-81, Pasadena, CA 91125-8100; 11 Department of Materials Science, The University of Tennessee Knoxville, TN 37996-2100.

## Abstract

Slowly-compressed single crystals, bulk metallic glasses (BMGs), rocks, granular materials, and the earth all deform via intermittent slips or “quakes”. We find that although these systems span 12 decades in length scale, they all show the same scaling behavior for their slip size distributions and other statistical properties. Remarkably, the size distributions follow the same power law multiplied with the same exponential cutoff. The cutoff grows with applied force for materials spanning length scales from nanometers to kilometers. The tuneability of the cutoff with stress reflects “tuned critical” behavior, rather than self-organized criticality (SOC), which would imply stress-independence. A simple mean field model for avalanches of slipping weak spots explains the agreement across scales. It predicts the observed slip-size distributions and the observed stress-dependent cutoff function. The results enable extrapolations from one scale to another, and from one force to another, across different materials and structures, from nanocrystals to earthquakes.

When solid materials such as nanometer single crystals (nanocrystals), bulk metallic glasses (BMGs), rocks, or granular materials are slowly deformed by compression or shear, they slip intermittently with slip-avalanches similar to earthquakes (Fig. 1). Typically these systems are studied separately. Here we find that the scaling behavior of their slip statistics agree within statistical error bars across a surprisingly wide range of different length scales and material structures. Figure 2 shows the probability, *C*(*S, F*), of observing slips larger than size *S*, at large applied forces or stresses, *F*. (*C*(*S, F*) is also called the complementary cumulative distribution function (CCDF) of slips observed in a small window of stresses around stress *F*). Figure 2 shows the CCDFs for five different materials spanning length scales ranging from nanometers to kilometers. Surprisingly, the distributions follow the same power law for nanocrystals, BMGs, granular materials, rocks, and earthquakes.

However, in many systems, including nanocrystals^1^ and earthquakes^2^,^3^ the slip statistics change with the applied stress *F*. For example, the sizes of the largest slips typically increase with stress. Here we first discuss a simple model that predicts this stress dependence. The model predicts that it is the same across the various materials and length scales of Fig. 1. We then test this prediction experimentally/observationally using the 5 materials of Fig. 2, spanning about 12 decades in length scale. We find good agreement of the data with the model predictions. The model makes many additional predictions for future experiments.

Identifying agreement in aspects of the slip statistics is important, because it enables us to transfer results from one scale to another, from one material to another, from one stress to another, or from one strain rate to another. The study shows how to use the fluctuations in the stress strain curves to identify and explain commonalities in the deformation mechanisms of different materials on different scales. The results provide new tools and methods to use the slip statistics to predict future materials deformation. The results also clarify which system parameters relevantly affect the deformation behavior on long length scales. We expect the results to be useful for applications in materials testing, failure prediction, and hazard prevention.

The simple mean field model predicts the scaling behavior of the slip size distributions. For example around the highest applied stresses *F* that the materials can support, the CCDFs scale as *C*(*S, F*) ~ *S*^*−*(*κ−*1)^ for a wide size range 0 < *S* < *S*_*max*_ where *S*_*max*_ is a stress-dependent “cutoff” size, and the power-law exponent *κ* − 1 = 1*/*2 does not depend on the microscopic details of the material (*i.e.* it is *universal*)^4^,^5^,^6^,^7^. The predicted exponent is indicated by the slope triangle in Fig. 2. Clearly, this prediction agrees well with the shown experiments, irrespective of scale and material structure. The exponent κ is important for applications because it tells how many large slips or large earthquakes are expected on average. (If κ were smaller, more large slips would be observed, while if it were larger, mostly smaller slips would be seen.)

The model predicts many additional statistical and dynamical properties of the slip-avalanches that agree with the experiments, including how the size of the largest slips depends on stress (see Table 1, Figs 3 and 4, and references^4^,^5^,^6^,^7^,^8^,^9^).

The model makes remarkably few assumptions. It predicts that the materials’ microscopic details do not affect the slip statistics on long length and time scales, regardless of the scale of the system.

In the following we first summarize key assumptions and predictions of the model^6^,^7^ and then discuss their comparison to experimental data.

## Model assumptions and predictions:

The model assumes that solids have elastically coupled weak spots that slip easily. These weak spots can have different origins, such as dislocations in crystals^1^, or shear transformation zones (STZs) in bulk metallic glasses (BMGs)^8^. As a solid is sheared, each weak spot is stuck until the local shear stress exceeds a random failure threshold. It then slips by a random amount until it re-sticks. The released stress is redistributed to all other weak spots. Thus a slipping weak spot can trigger other spots to also slip in a slip avalanche, or “slip”. Using tools from the theory of phase transitions, such as the renormalization group, one can show that the slip statistics of the model do not depend on the details of the system. For example, the model predicts that the scaling behavior for the slip size distribution neither depends on the details of the distribution of stress thresholds, nor on the details of the triggering mechanism^10^. We assume the shear rate is slow, such that each slip-avalanche is completed before a new one is started. The predictions for the slip statistics of the model agree with recent experiments on *ductile* materials, such as single crystals^1^,^11^ and high entropy alloys^12^,^13^.

By adding threshold weakening to the model, it can also describe *brittle* materials and materials that show *stick-slip* behavior, such as bulk metallic glasses^8^,^9^, granular materials, and rocks. Threshold weakening means that the local failure stress of a weak spot weakens when that weak spot slips in an avalanche. Weakening may model dilation, local softening due to heating, or fracture effects in bulk metallic glasses, rocks, and granular materials. The weakened thresholds are assumed to re-heal to their original strength during the times between avalanches. For small weakening our model predicts randomly occurring small slips with a broad distribution of sizes, mixed with almost-periodically recurring, much larger (system-spanning) slips, whose sizes are narrowly distributed. In the following we focus on the broadly distributed (“small”) avalanches in *brittle and in ductile* materials, and remove the almost-periodically recurring largest events from the analysis.

First, we briefly summarize the mathematical description of the model. Both the continuum and the discrete versions of the model give the same results for the serration statistics^5^,^6^,^7^. In the discrete version the weak spots are put on *N* lattice points. The local stress*, τ*_*l*_, at a lattice point, *l*, for applied shear stress (*F*) is given by^5^,^6^,^7^: 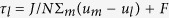, where *J/N* is the elastic mean field coupling between weak spots, *u*_*l*_ is the cumulative slip of a weak spot, *l,* in the shear direction. Using the renormalization group, one can show that on long length scales the simple mean field coupling *J/N* leads to the same slip statistics as the physical long-range elastic interactions *J*(*r*) between local slips *u*_*l*_, that typically decay with distance *r* as *J*(*r*) ~ 1*/r*^3^ or slower^5^,^6^,^7^. For slow strain-rate loading conditions, the applied stress is replaced by *F* = *K*_*L*_(*vt* − *u*_*l*_) where *t* is time, *K*_*L*_ is a weak spring constant, modeling the coupling of weak spot *l* to the moving sample boundary, and *v* is the speed at which the boundary moves in the shear direction.

Each weak spot fails when the local stress, *τ*_*l*_, is larger than the local static failure threshold stress, called *τ*_*f,l*_ = *τ*_*s,l*_, or its dynamically weakened value *τ*_*f,l*_ = *τ*_*d,l*_^14^,^15^. The (static or dynamic) threshold stresses, *τ*_*f,l*_, are narrowly distributed. When the weak spot at site *l* fails, it slips by a certain amount, Δ*u*_*l*_, thereby relaxing the local stress by *τ*_*f,l*_ − *τ*_*a,l*_ ~ 2*G*Δ*u*_*l*_, where *G* ~ *J* is the elastic shear modulus, and *τ*_*a,l*_ is a random local arrest stress, also taken from a narrow probability distribution. The released stress is equally redistributed to the other weak spots in the system. This stress redistribution may trigger other spots to slip, thus leading to a slip avalanche, which is measurable as a serration in the stress-strain curve or as acoustic emission. More details and the model and its analytic solution are given in^5^,^6^,^7^.

We purposefully focus on the predictions of the simplest version of the model that accounts for the key features of the statistics observed in the experiments reported here. The simplicity of the model allows for an analytic solution, which helps to build an intuition for the underlying physics, to organize the data, and to identify the key experimental tuning parameters that relevantly affect the slip statistics in our experiments.

(Simulations of more complex models that account for additional effects, such as pore pressure if fluids in the solid are affecting the slip statistics, give similar, though more complex behavior^16^,^17^.)

### Model predictions for the avalanche statistics

The model predicts the slip size distributions for experiments where the stress is slowly increased or where the sample is deformed at a slow strain-rate. The model predicts that the slip size distribution follows a power law that extends to a maximum size *S*_*max*_ that changes with applied stress or imposed strain-rate. For example, for large avalanche sizes *S* the stress-dependent CCDF is predicted to scale as


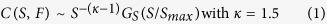


and scaling function


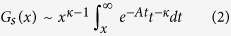


with a material dependent constant *A*^18^, see Table 1a^6^,^7^. Table 1b shows the quantitative dependence of *S*_*max*_ on applied stress *F* (for load-controlled experiments) or imposed strain-rate *Ω* (for displacement-controlled experiments). For example, for load controlled experiments, in the absence of work-hardening, the cutoff increases with stress as


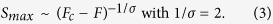


Here *F*_*c*_ is the maximum flow- or failure-stress of the material. The cutoff *S*_*max*_ is largest for stress *F* near the maximum stress *F*_*c*_. This implies that the largest range of power law scaling of the slip size distribution *C*(*S, F*) is observed for *F* near *F*_*c*_. For this reason in Fig. 2 we show slip size distributions for stress windows near the maximum stress in each system.

Similar results are shown in Table 1b for the CCDFs *C*(*T*) of the slip durations *T*, *C*(*V*), of the slip-avalanche propagation rates, (measured as stress drop rates *V* during a slip-avalanche), and the power spectra *P*(*ω*) of the stress drop rate time traces *V*(*t*). Here the power spectra are defined as the absolute square of the Fourier transform of the slip-avalanche propagation rates^8^.

### Tools for testing model predictions against experimental data

Equations (1) and (2) imply that far below the failure stress *F*_*c*_, the slip size distribution, *C*(*S, F*) drops off much more steeply than at *F*_*c*_. If at these low stresses *C*(*S, F*) is naively fitted with a power-law, then the stress dependent cutoff *S*_*max*_ given by Equation (3) can cause the fitted power-law exponents to falsely appear as if they would depend on the applied stress^19^,^20^, see Fig. 3. Usually distributions at the highest stresses are expected to give the best estimates of the correct scaling exponent, as shown in Fig. 2. Widom scaling collapses^1^,^21^,^22^,^23^ of *C*(*S, F*) at different stresses however constitute an even stronger test of the theory than mere power-law fits at the highest stresses. They not only yield the correctly extrapolated universal scaling *exponents* but also the cutoff *scaling-functions*, by accounting not only for the power-law distributions but also for their tunable cutoffs^20^. For nanocrystals, a scaling collapse analysis for slip-avalanche size distributions is given in^1^. Additional collapses for bulk metallic glasses in^8^,^9^ show strong agreement with the model predictions for twelve different statistical and dynamical quantities. Below we use a new scaling collapse for data on nanocrystals, BMGs, rock friction, granular materials, and earthquakes (Fig. 3) that is designed for directly comparing the slip statistics of different systems.

### Collapsing data of different systems using the fourth moment of the avalanche size distribution

Directly comparing data collapses from experiments on different systems can be difficult because often the critical stress *F*_c_ is unknown or only approximately known. One way around this difficulty is to replace tuning parameters, such as stress *F*, with moments of the probability density function 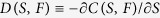 of slip-sizes *S* in a stress bin around *F*. Here we use the 4^th^ moment 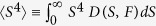 for the scaling collapses, because it strongly depends on the largest avalanche size, and thus on the stress *F*.

By plugging the relationship between the 4^th^ moment and the stress 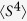 ~ (*F*_*c*_*–F*)^(*k−*5)*/σ*^ into the scaling form for 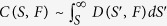 derived in^1^, the model predicts the scaling form


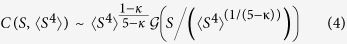


where *κ* = 3*/*2 is the universal exponent and


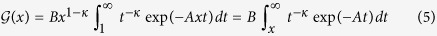


is a universal scaling-function. The constants *A* and *B* are non-universal, i.e. they differ for each material. Note that Equation (4) no longer requires knowledge of the stress *F*. It is especially useful for comparing data from systems where the exact value of *F* is unknown. Equation (4) implies that the size of the cutoff scales with the fourth moment as


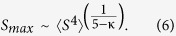


In the following we test these model predictions against experimental data.

## Comparison to experiments

Figure 2 illustrates the striking agreement between model predictions and experiments and observations on sheared nanocrystals, bulk metallic glasses, rocks, jammed granular materials, and earthquakes. Even though the systems span 12 decades in length scale, the CCDFs follow the predicted power-law *S*^*−*1*/*2^, over the entire plotted range of sizes.

Table 1c shows excellent agreement for many additional statistical distributions obtained from the model and experiments on the same systems. Remarkably, the experiments and model agree not only in the power-law exponents but also in the in the cutoff dependence on stress^1^ or strain-rate^9^. They also agree with high time-resolution properties of the slip *dynamics*, such as power spectra and temporal avalanche shapes^8^. Empty entries in the table highlight additional model predictions for future high-resolution experiments.

## Scaling collapses for experimental and observational data

### Nano-single-crystals

Jennings, Greer and collaborators recorded slip statistics in seven slowly compressed Mo-nano-single-crystals with approximate diameter of 800 nm, compressed at nominal displacement rate of 0.1 nm/s. The details of the experiments are given in^1^ and the Methods Section. On average, the data show larger slips for higher applied stresses^1^, in agreement with our model predictions.

### Bulk metallic glasses

Gu, Hufnagel, and Wright studied a composite material with overall composition 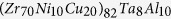, (atomic %), which is a bulk metallic glass matrix reinforced by ~20 micron-scale Ta-rich solid solution particles^15^. Cylinders with lengths of 8.1 mm and a 3:1 aspect ratio aspect ratio were slowly compressed at a strain rate of 10^–3^ s^–1^ (see Methods Section for details), measuring the applied stress at a frequency of 100 kHz. Larger stresses lead to larger slips, in agreement with our model predictions^9^.

### Rocks

Goebel, Schorlemmer, Becker, and Dresen recorded acoustic emission data for stick-slip events in slowly compressed rocks. Details of the experimental setup are given in^19^,^24^,^25^,^26^. The acoustic-emission statistics show that *b*-values decrease in the periods just before the almost-periodically recurring large slips. A decrease in the *b*-value is consistent with observing larger slips. The stress is generally the largest when the largest slips are observed. The compressed rocks show more large slips for higher stresses, in agreement with our model predictions.

### Granular materials

Denisov, Schall, and collaborators shear granular materials in a cuboid shear-cell setup that provides force measurement by built-in pressure sensors. This setup makes it possible to track fluctuations of the applied force with good time resolution while imaging the internal strain distribution over the full granulate volume (see Methods). Application of a load puts the granulate under constant external pressure. The granulate is sheared uniformly at constant (low) shear rate of dγ/dt = 3.6 × 10^*−*4^ s^−1^ starting from a well-defined initial state up to a strain of 0.2. To increase statistics, we average over ten shear experiments. Again, larger stresses lead to larger slips, as predicted by the model.

### Earthquakes

Frequency-magnitude distributions at different rake angles for earthquakes observed in Southern California^2^,^3^ showed that the *b*-value (related to our model exponent via *b* = 3(*κ* − 1)*/*2) strongly depends on the rake angle of the associated fault type. (The rake angle describes the direction of the fault motion with respect to the orientation of the fault.) Schorlemmer *et al.*^2^,^3^ showed that the rake angle is directly related to the stress on the fault, thereby relating the *b-*value to the stress on the fault. The data showed that smaller *b-*values (indicating more large earthquakes) are observed for higher stresses on the fault, (with the highest stresses corresponding to rake angles near 90 degrees). The correlation of increasing earthquake size and smaller *b*-values for increasing stress agrees with our model predictions.

### Comparison

To quantitatively test the model predictions against the experiments and observations, the slip-size distributions for different stress windows were extracted from the data, as described in the Methods Section. For each stress interval, 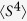 is computed from the slip-size distribution. The distributions 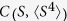 for each value of 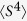 are shown in the main parts of Fig. 3A–F. In Fig. 3G–L all distributions are collapsed onto each other according to Equations (4) and (6) by plotting 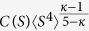 against 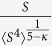 for the predicted exponent *κ* = 3*/*2. Figure 4 shows the agreement of the scaling collapse functions with each other and with the overlaid predicted scaling function of Equation (5) (in grey). Figure 4 thus constitutes a strong confirmation of the agreement of the five data sets spanning 12 decades in length: The data sets agree with each other and with the mean-field model predictions for both the predicted scaling exponents and the scaling function. The top part of Fig. 4 shows that the collapses for nanocrystals, bulk metallic glasses, rocks and earthquakes closely follow the predicted scaling function *G*(*x*) of Equation (5) above, for the predicted scaling exponent κ = 1.5. The lower part of Fig. 4 exhibits a slight deviation in the scaling function for the granular data from the predicted scaling function. The reason lies in the deviation of the packing fraction in the granular experiments from random closed packed. (The packing fraction in the experiments is about 90% of random closed packed, while the scaling function *G*(*x*) from Equation (5) is predicted for experiments at random closed packed packing fraction^7^.) More details are given in the Supplementary Information.

## Interpretation of the Results

The many agreements between model predictions and experiments evidenced in Table 1c and Figs 2, 3, 4 suggest far-reaching universality in these systems. Experiments and observations agree (within statistical error-bars) with predictions^6^,^7^,^21^ that the length scale and material structure do not affect the scaling properties of the slip-avalanche statistics. Consequently, universal scaling exponents and functions for slip statistics apply across a wide range of length scales, from nanocrystals, to bulk metallic glasses, to granular materials, to rocks, to earthquakes. Interesting connections to an even larger range of systems include close similarities with slowly deformed ferroelastics^27^ and porous materials^28^. The model quantitatively predicts how the largest (“cutoff”) slip-size *S*_*max*_ increases with stress as the failure stress is approached. The present study provides many additional predictions (see Table 1c), for future experiments and simulations.

The model and the experiments provide a unified understanding of intermittent deformation. The experiments show that the model can be used to identify and predict trends in the slip statistics that are independent of the microscopic details of the system. (The agreement of the scaling properties of the slip statistics across scales does not imply the predictability of individual slips or earthquake events. Rather it implies that we can predict the scaling behavior of average properties of the slip statistics and the probability of slips of a certain size, including their dependence on stress and strain-rate.)

The results are useful to help organize new experimental data, to predict the slip statistics at high stresses from that observed at lower stresses, and to transfer results across a wide range of materials and length scales. They also provide a quantitative basis for further applications, such as materials testing on a wide range of laboratory scales and hazard prediction studies of earthquakes on much larger, tectonic scales.

## Methods

### Data-analysis methods

Details of the data analysis are given in the Supplementary Information.

**High stress bin cumulative distributions for** Fig. 2: The MFT model predicts that at near-maximum stresses, near *F*_*max*_ (and consequently for the highest 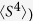, the cumulative slip-size distribution follows a power-law, *C*(*S, F*_*max*_) ~ *S*^*−*1*/*2^ over a wide range of sizes^1^. Because the earthquake data had relatively few events, the quarter of the data at the highest stresses were used. For nanocrystals, BMGs, and rocks data in the 80–85% stress range were used, and for granular materials data in the 65–70% stress range were used. (For additional ranges of the granular data see Supplementary Information). This choice ensured that for each experiment we used the largest stress values, where the avalanches were large but still smaller than the sample size, so that the distributions were not distorted by finite sample size effects^1^.

In Fig. 2 the power-law scaling regimes of the resulting CCDFs were collapsed onto one another by multiplying the *x*- and *y*-axes with different constants *k*_*x*_ and *k*_*y*_ to account for the difference of scales and units in the different systems. The values for these rescaling constants are given in Table 2 below. (Fig. 2 only shows the data in the scaling regime of the distributions. For the complete data range see Supplementary Information.)

## Experimental Methods:

For rocks^19^,^24^,^25^,^26^ and earthquakes^2^,^3^ the measurement techniques are described in the corresponding references given in the text. For the rocks we assumed that the acoustic emission amplitude (measured in mV) is proportional to the moment of the slip that created the acoustic emission. This assumption is supported by the model predictions for the slips that we consider here, *i.e.* for the slips that are within the power-law regime of the slip-size distribution^4^.

Details on the nanocrystal experiments of Fig. 2 are given in^29^. Uniaxial compression tests in a G200 Nanoindenter (Agilent Technologies) were performed using the dynamic-contact module (DCM) fitted with a 7 micron-diameter diamond flat punch. Each compression test was conducted under a nominal constant displacement rate of 0.1 nm/s. Compression tests were performed on 7 single-crystalline, cylindrical Mo nano-single-crystals with diameters of 800 nm and aspect ratios (height/diameter) of 6:1. The nanocrystals were prepared using a focused ion beam (FIB) on well-annealed electropolished (100) crystals^27^,^28^. (Note that in general single crystals are better suited for studying scale free behavior on a wide range of scales than polycrystalline materials, because they are free from interfering grain size effects^11^,^30^.

For the BMG experiments, a metallic-glass-matrix composite (MGMC) was used, with an overall composition of (Zr_70_Ni_10_Cu_20_)_82_Ta_8_Al_10_ (atomic percent). The composite consists of 10–30 μm particles of a ductile Ta-rich solid solution with a body-centered cubic structure embedded in a Zr-based metallic glass matrix^15^. Cylindrical 3 mm-diameter rods of the composite were produced by arc-melting master alloy ingots and suction casting into a copper mold. The diameters of the cast rods were reduced to 2.7 mm using centerless grinding. The cylinders were cut to nominal lengths of 8.1 mm using electrode discharge machining. The ends of the compression specimens were polished to a parallelism within 1.5 μm. An Instron 5584 mechanical test system was used to compress these specimens at a nominal strain rate of 10^–3^ s^–1^. The load data were acquired with a piezoelectric load cell (Kistler 9031A) and charge amplifier (Kistler 5010B) at 100 kHz using a Hi-Techniques Synergy P data acquisition system. The lowest frequency of the low pass filters in the data acquisition system was 40 kHz. Custom fixturing ensured uniaxial loading of the specimen as well as a high load frame stiffness. The data from the fracture event was used as the unit impulse response for Wiener filtering.

**Figure 1:**
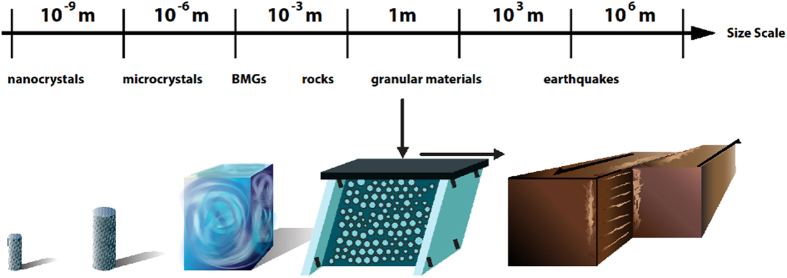
Sketch of size scales of samples, spanning 12-13 decades in length, and showing the same slip-avalanche statistics, as summarized in Figures 2,3,4 **and** Table 1. (Figure courtesy of Matthew Brinkman.)

**Figure 2:**
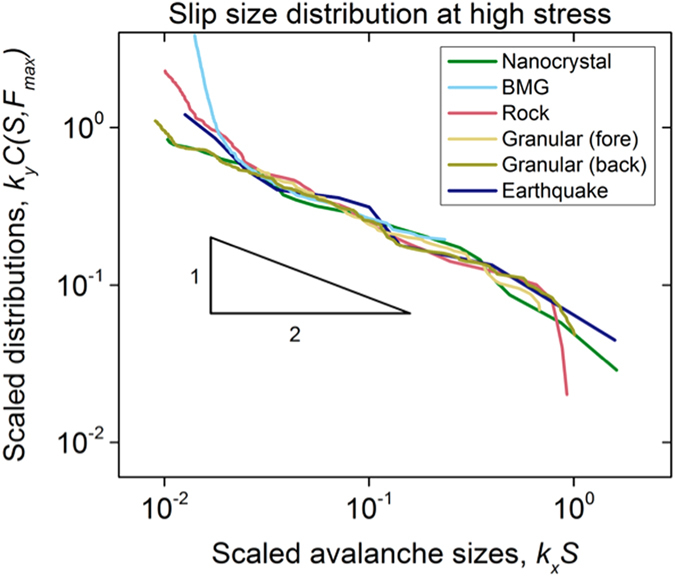
Probability. *C*(*S*, *F*_max_) of observing slip sizes larger than size *S* in a stress-bin near maximum applied stresses *F*_max_ for slowly-compressed nanocrystals (green), bulk metallic glasses (BMGs) (turquoise)^8^,^9^,^18^, rocks (red), granular materials (yellow and light green), and earthquake data (purple)^2^,^3^. (For rescaling constants *k*_x_ and *k*_y_, see Methods section and Supplementary Information). They follow the predicted power-law of −1/2 (triangle). Slip-size ranges: 0.4514–64.9168 nm (nanocrystals), 0.1450–4.4376 MPa (BMG stress-drops), 1010 − 9.2629 × 10^4^ mV (rock friction acoustic emission amplitudes), 0.0091–1.3851 N (granular materials force-drops, forward shear), 0.0573–1.6689 N (granular, backward shear, measured with a different instrument), 4.4601 × 10^14^–5.6234 × 10^16^ Nm (earthquake moments, Southern California).

**Figure 3:**
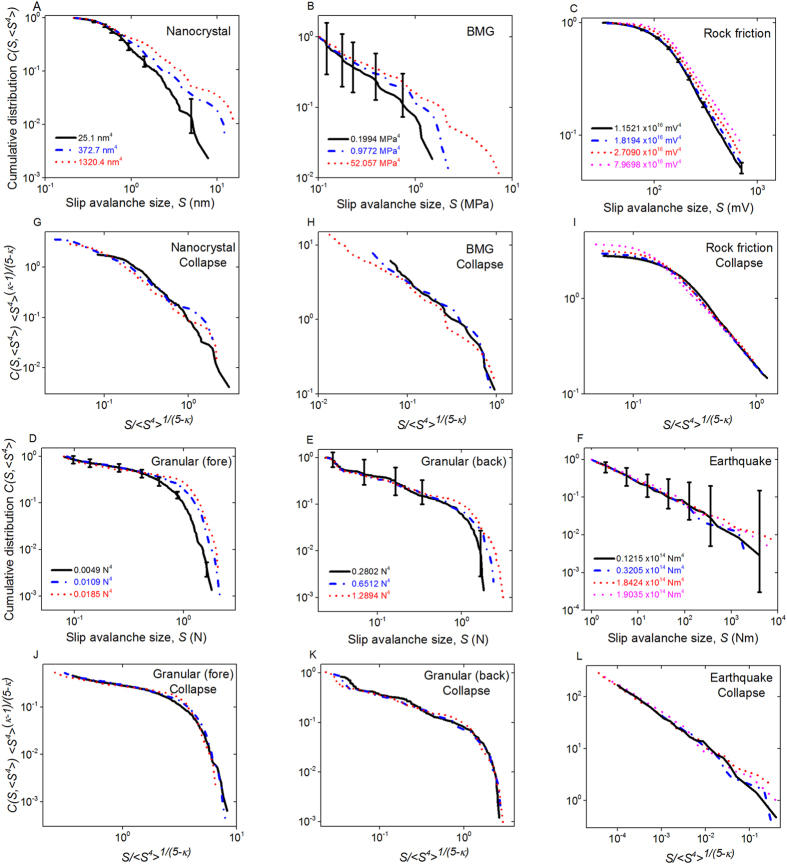
(**A–F**) Complementary cumulative slip-size distributions for the five data sets. Legends give the 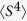 values for the logarithmic bins in 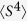 (see Methods Section). Granular (Fore) represents the distributions for forwards shear experiments, and Granular (Back) represents the distributions for the backwards shear experiments that used a different measurement instrument. (**G–L**) Scaling collapses, each plot collapsing the CCDFs above it, by rescaling x and y axes with the shown powers of 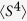, see text. The power-law exponents agree with the mean field theory predictions in Equation (4) in the text. κ = 1.5 has a 10% error bar (or smaller).

**Figure 4:**
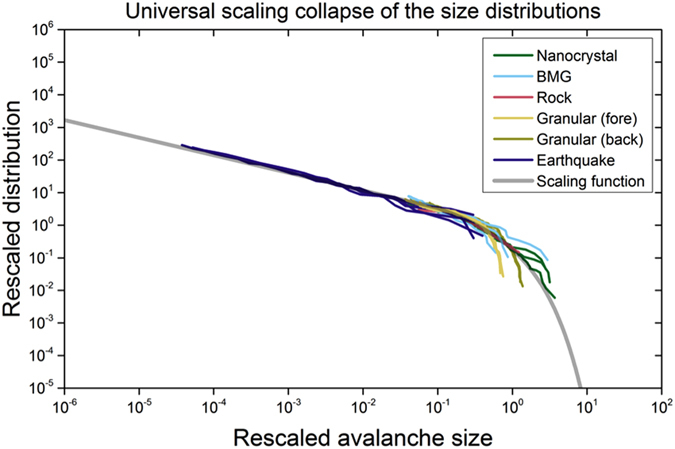
Scaling collapses of Figure 3G–L plotted upon the theoretically predicted scaling function of Equation (5) , G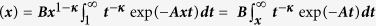. A and B are non-universal constants. All collapses use the mean field exponent *κ* = 1.5. While the five systems span 12 decades in length, from earthquakes down to nanocrystals, all rescaled CCDFs fit onto the same scaling function predicted by our theory. Statistical fluctuations due to lower event numbers cause the slight deviation for the largest slips. For the granular data, it is in part caused by a packing fraction that is below random closed packed (at about 90% of random closed packed) during the granular shear experiments (see Supplementary Information).

For the slow shear of granular materials, Denisov, Schall, and collaborators used polymethyl methacrylate (PMMA) spheres^31^ with diameter of 1.5 mm and polydispersity of ~5%. The particles were filled into a cuboid shear device with transparent, tiltable side walls with built-in pressure sensors (see Fig. 1). The shear cell had dimensions of 10 × 10 × 10 cm^3^, containing about 3 × 10^5^ particles. A top plate charged with additional weights was used to confine the granulate vertically, exerting a constant normal force between 10 and 100 N on the top layer of the granulate. After a fixed pre-shear protocol generating a reproducible initial packing, the granulate was sheared at a constant strain rate dγ/dt = 3.6 × 10^*−*4^ to a total strain of *γ* = 20%. The applied force is measured at a frequency of 500 Hz with an accuracy of ±10^−2^ N, and a maximum force on each sensor of 45 N.

## Additional Information

**How to cite this article**: Uhl, J. T. *et al.* Universal Quake Statistics: From Compressed Nanocrystals to Earthquakes. *Sci. Rep.* **5**, 16493; doi: 10.1038/srep16493 (2015).

## Supplementary Material

Supplementary Information

## Figures and Tables

**Table 1:** 

(a)							
Statistical Distributions	Scaling forms predicted by the model^5^,^6^,^7^						
CCDF, *C*(*S*), of avalanche size, *S*	*C*(*S*) ~ *S*^*−*(*κ−*1)^ *G*_*S*_(*S/S*_*max*_)						
CCDF, *C*(*V*), of stress-drop rate, *V* ~ *S/T*	*C*(*V*) ~ *V*^*−*(*ψ−*1)^ *G*_*V*_(*V/V*_*max*_)						
CCDF, *C*(*T*), of avalanche durations, *T*	*C*(*T*) ~ *T*^*−*(*α−*1)^*G*_*T*_(*T/T*_*max*_)						
Power spectrum, *P*(*ω*), at frequency, *ω*	*P*(*ω*) ~ *ω*^*−φ*^*D*_*ω*_(*ω/ω*_*min*_)						
(b)							
**Fixed-stress loading conditions: slowly increasing stress,** ***F,*** **up to the failure stress,** ***F***_***c***_	**Fixed-strain-rate loading conditions: moving the boundary at a slow strain rate,** ***Ω***						
*S*_*max*_ ~ (*F*_*c*_ *−* *F*)^*−*1*/σ*^	*S*_*max*_ ~ *Ω*^*−φλ*^						
*V*_*max*_ ~ (*F*_*c*_ − *F*)^*−*(1*+ρ*)*/*(*σφλ*)^	*V*_*max*_ ~ *Ω*^*−*(1*+ρ*)^						
*T*_*max*_ ~ (*F*_*c*_ − *F*)^*−*1*/*(*σφ*)^	*T*_*max*_ ~ *Ω*^*−λ*^						
*ω*_*min*_ ~ (*F*_*c*_ *−* *F*)^1*/*(*σφ*)^	*ω*_*min*_ ~ *Ω*^*λ*^						
(c)							
**Exponents**	**Sample Sizes**	***κ***	***κ + σ***	***σ***	***φ***	***α***	***ψ***
**Model Predictions**							
Mean Field Theory (MFT)^7^		**1.5**	2	0.5	2	2	1
Experimental Verifications*							
Nanocrystals (Molybdenum (Mo), Compression, see^1^,^29^ and Figs 2, 3, 4)	10^−8^ m	**1.5**	2	0.5	2		
Microcrystals (Nickel (Ni), Compression^32^,^33^)	10^−6^ m	**1.5**			2		
Bulk Metallic Glass (BMG) (Cu47Zr47.5Al5^18^, Zr45Hf12Nb5Cu15.4Ni12.6Al10^8^, and Zr64.13Cu15.75Ni10.12Al10^9^, atomic percent) Compression.	10^−3^ m	**1.5**	2	0.5	2	2	
Lab-scale rocks (Sidobre granite, Compression^19^,^20^,^34^)	10^−2^ m	**~1.5**	1.66–2.2				
Lab-scale rocks (Westerly granite, Frictional sliding^24^)	10^−2^ m	**~1.5**					
Jammed granular materials (Photo-elastic disks in Couette cells and other geometries^7^)	1 m	**~1.5**			1.8–2.5	~2	
Earthquakes^4^,^14^,^35^	10^5^ m	**~1.5**			2		

Table 1. (a) Model predictions for scaling forms of various distributions.

Here G_S_(S), G_V_(V), G_T_(T), and D_ω_(ω) are universal scaling functions, κ, ψ, α, φ, σ, λ, and ρ are universal power-law exponents^7^, and S_max_, V_max_, T_max_, and ω_min_ are the cutoffs of the power-law regimes of the corresponding distributions^6^,^7^.

(b) Predicted scaling forms of the cutoffs for two loading-conditions, near failure^6^,^7^.

(c) Comparison of model (Mean Field Theory (MFT)) exponents with different experiments, showing strong agreement. Open entries indicate predictions for future experiments. MFT predicts that λ = 1 and ρ = 1^7^. Our experiments reveal an exponent of κ = 3/2 for nanocrystals down to 75 nm in size. Additional predictions are given in the SI.

*Exponents from experiments and observations quoted throughout this paper have a 10% error range due to statistical fluctuations. As shown in Figure 3A–C for compressed nanocrystals, bulk metallic glasses (BMGs), and rocks, power-law fits for small stresses (where the cutoff is small) would yield wrong exponent values, because those are skewed by the small exponential cutoff, as predicted by our model. Instead, scaling collapses like those of Figure 3G–L yield the correctly extrapolated exponents, which agree with our model predictions. Exponents from previous experiments were obtained from^19^,^20^ at the largest stresses, using that the Gutenberg Richter exponent, b, in^19^ is related to our exponents via b = 3(κ − 1)/2 (see^5^,^34^). For the relationship between the slip-size and the acoustic-emission signal see^34^, the Supplementary Information of^1^,^36^, and references therein.

**Table 2:** Values of the rescaling factors *k*_*x*_ and *k*_*y*_ used in Figure 2.

Material	*k*_*x*_	*k*_*y*_
Nanocrystal	1/40 nm^−1^	1.5
Bulk Metallic Glass (BMG)	1/10 MPa^−1^	22
Rock Friction	10^−5^ mV^−1^	45
Granular (Fore)	1 N^−1^	1.56
Granular (Back)	½ N^−1^	0.66
Earthquakes	1/(5000 * 7.0795 × 10^12^) N^−1^m^−1^	11
