# Supplementary material for: Universal Quake Statistics: From Compressed Nanocrystals to Earthquakes
Source: Sci Rep. 2015 Nov 17;5:16493. doi: 10.1038/srep16493 (PMC4647222; doi:10.1038/srep16493)
Supplement: Supplementary Information [file srep16493-s1.doc]

Supplementary Information:

Universal Quake Statistics: From Compressed Nanocrystals to Earthquakes

Jonathan T. Uhl^1^, Shivesh Pathak^2^, Danijel Schorlemmer^3,4^, Xin Liu^2^, Ryan Swindeman^2^, Braden A.W. Brinkman^2^,, Michael LeBlanc^2^, Georgios Tsekenis^2^, Nir Friedman^2^, Robert Behringer^5^, Dmitry Denisov^6^, Peter Schall^6^, Xiaojun Gu^7^, Wendelin J. Wright^7,8^, Todd Hufnagel^9^, Andrew Jennings^10^, Julia R. Greer^10^, P.K. Liaw^11^, Thorsten Becker^4^, Georg Dresen^3^, and

Karin A. Dahmen^2*^

**I. Extracting slip-avalanches:**

The data analysis for each of these experiments is performed analogously to the data analysis for the nanocrystals given below.

*Data Analysis*: The time series of the measured height *z(t)* of the nanocrystal as a function of time *t* was numerically differentiated to obtain *V(t) ≡ d(z(t))/dt*. A slip begins at time *t_1_,* and ends at time *t_2_*, if the following conditions are fulfilled, with the mean displacement rate *V_thr_*:

*V*(*t_1_*) *= V*(*t_2_*) *= V_thr_* and

*V*(*t*) *> V_thr_* for all *t* with *t_1_ < t < t_2._*

The size of the slip is *S ≡ z*(*t_2_*) *- z*(*t_1_*). This method is consistent with related analyses in [*37*].

For the analysis of the analytic model, the mean-field theory and standard tools from the theory of phase transition and the renormalization group were applied, as described in [*5-7*].

**II. Including the entire data range in Figure 2:**

**
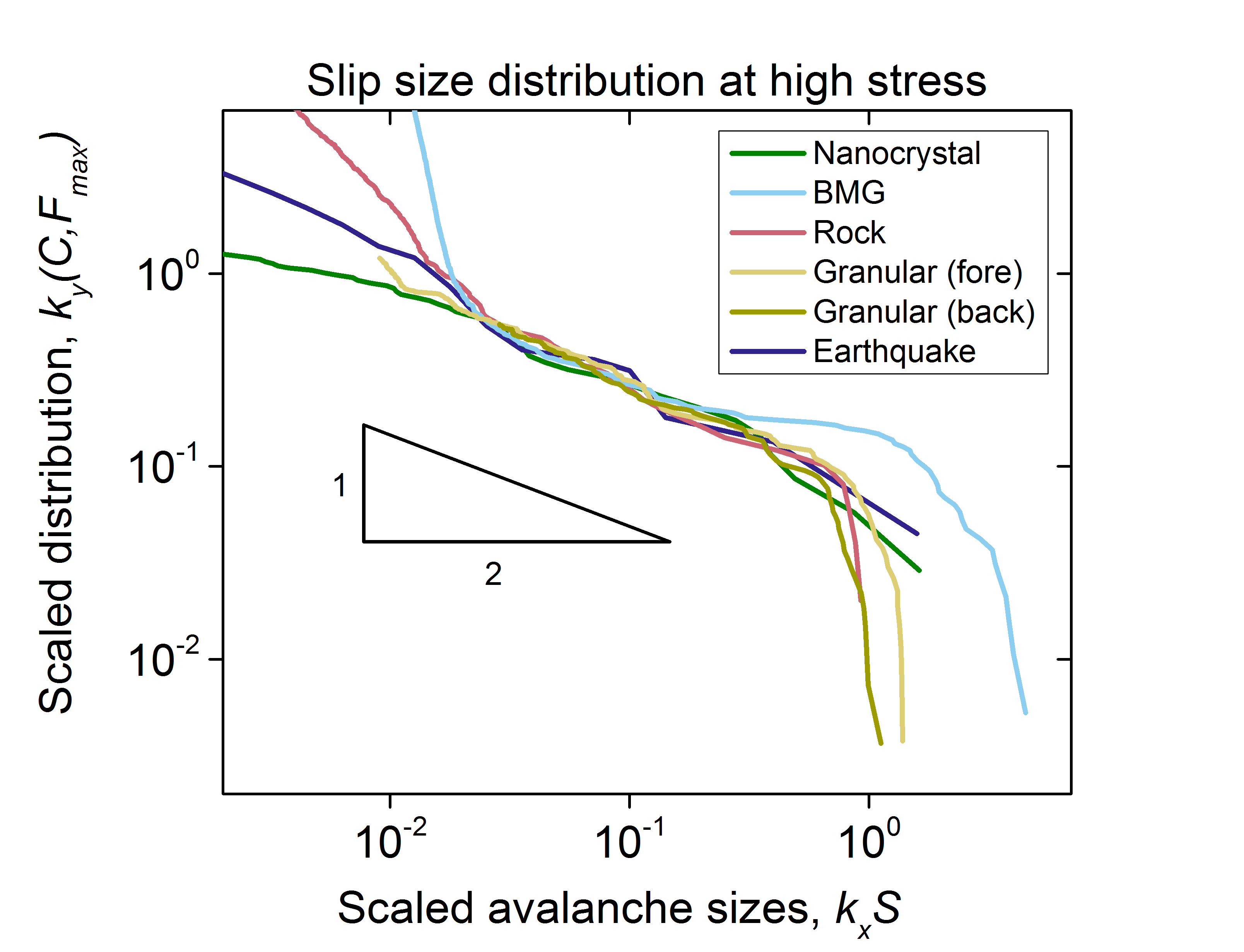
**

***Figure S1:*** *Same content as Figure 2 in the main paper, but showing the entire range of available data. The bulk metallic glass (BMG) data shows a bump at the end, which reflects the almost periodically recurring large slips, often seen in stick slip data. The slips for the nanocrystals range from 0.0033 to 64.9168 nm, the stress drops in the BMG from 0.1450 to 73.3327 MPa, the amplitude drops in the rocks from 21.95 to 9.2629*
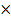
*10^4^ mV, the force drops in the granular material from 0.0091 to 1.3851 N (fore) and 0.0573 to 2.2469 N (back), and the earthquake moments from 7.0795*
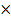
*10^12^ to 5.6234*
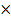
*10^16^ Nm. Further, scaled avalanche sizes that have magnitude < 10 ̶ 2 have been removed from the above figure. They are irrelevant for the analysis of the scaling behavior on long length scales. The largest and the smallest slips do not belong to the scaling regime described in the paper, see reference [8], and were therefore omitted from Figure 2 in the main paper.*

***
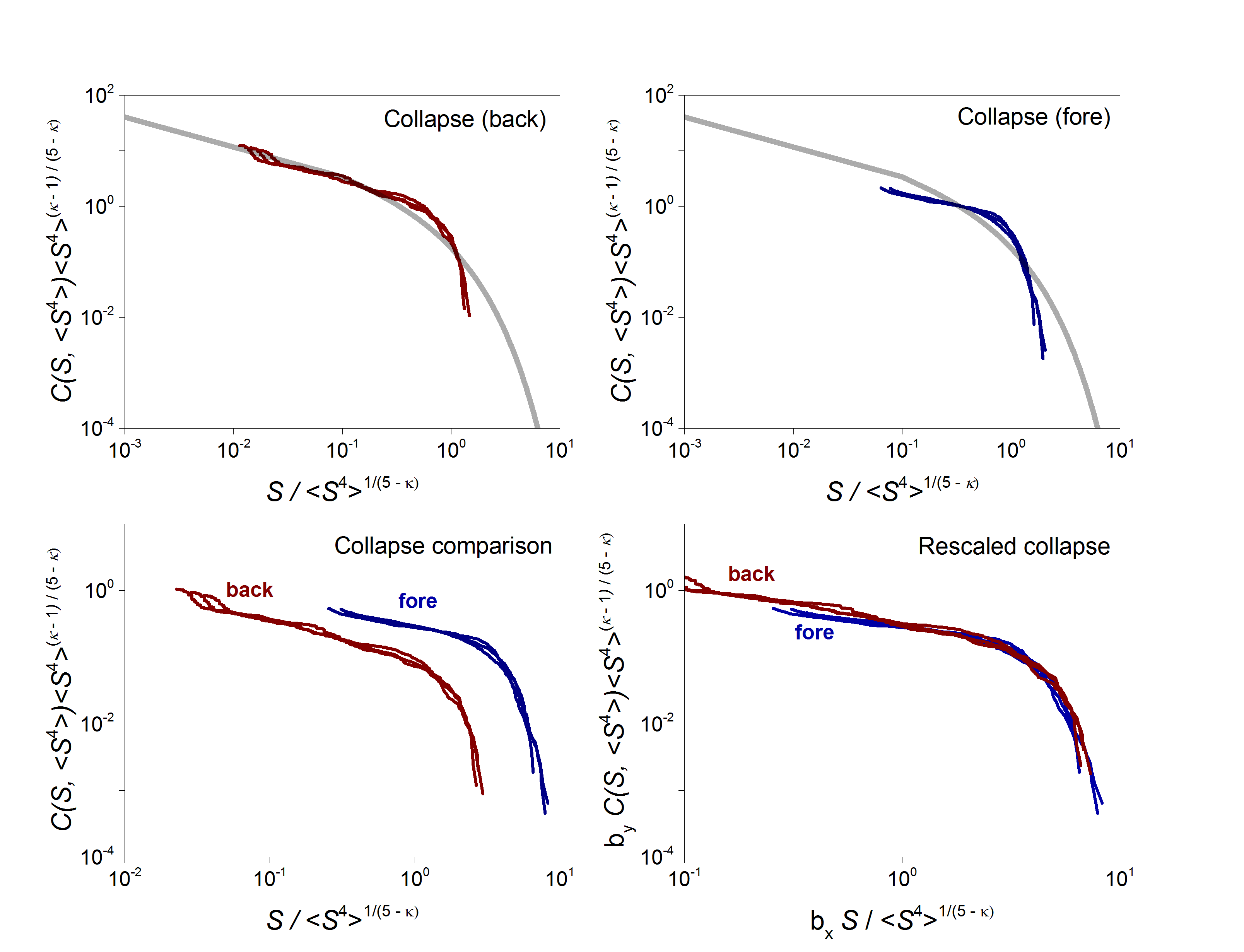
***

***Figure S2:*** *The granular data is peculiar since the experiments were run in two different ways: 5 experiments were run shearing the top of the load cell in Figure 1 in the main paper in a forwards direction and 5 experiments shearing it in a backwards direction. The instruments measuring the stress time series were different for the forward and backwards direction. The plots (going clockwise from upper left) represent: the scaling collapse of the backwards experiments onto the scaling function G(x) of Equation (5) and Figure 4 in the main text; the scaling collapse of the forwards experiments onto the same scaling function; a combined collapse, where the scaling collapses are shifted to fall on top of each other – in order to do this, the x- and y-axes of the backwards data were multiplied by b_x_=2.5 and b_y_=2 respectively; and finally, a comparative collapse showing the relative difference in magnitude of the events between the forwards and backwards experiments, resulting from the different instruments that were used. Figure S3 below shows how finite size effects can be removed from the collapses.*

**
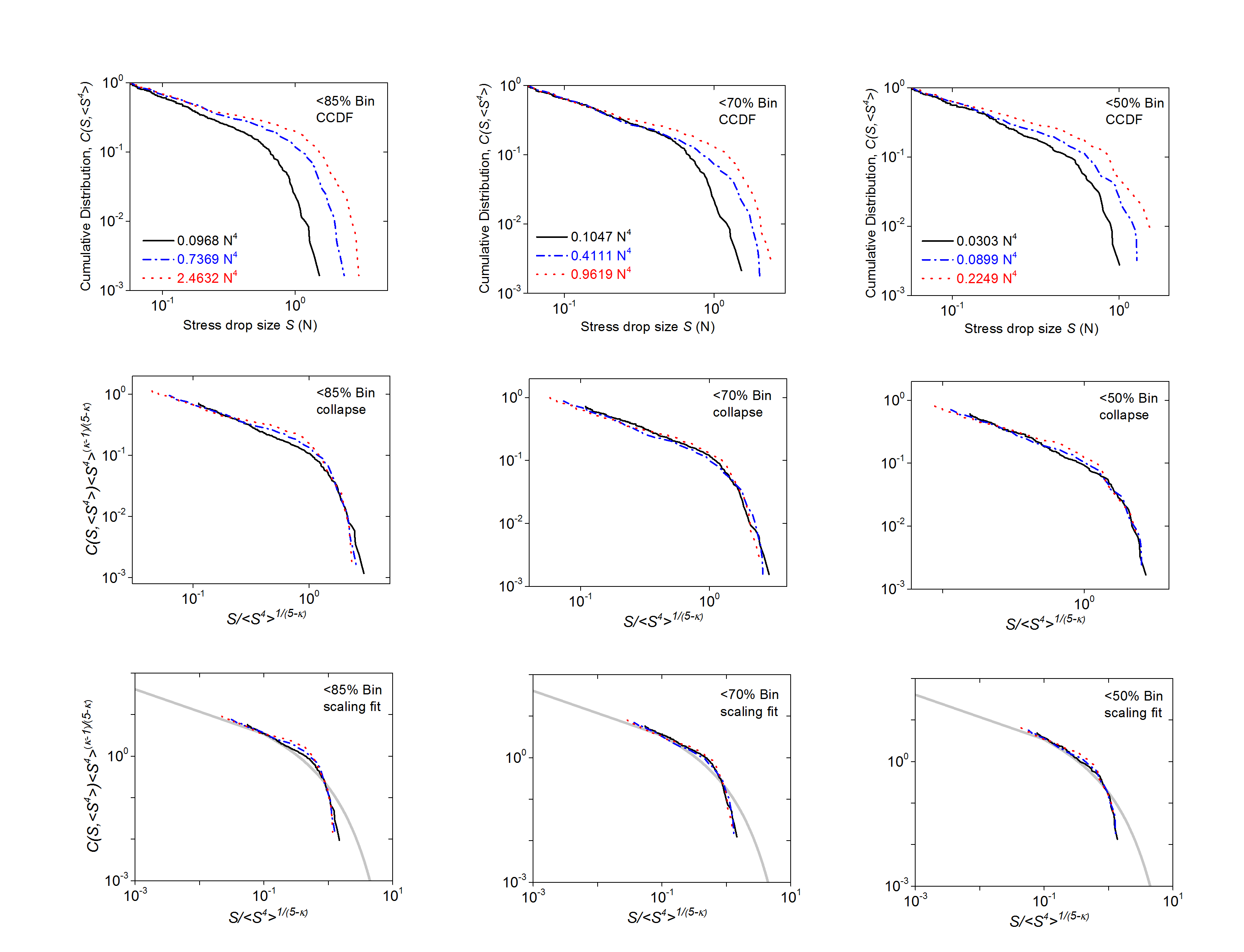
**

***Figure S3:*** *The first row shows the cumulative distributions (CCDFs) of events from the backwards granular data, in a specific stress range. The numbers in the legends again signify the average <S^4^> values for the logarithmic bins in <S^4^> (see Methods Section in the main text). The first column uses data at stresses smaller than 85% of the maximum stress, the second column at stresses less than 70%, and the third column at stresses less than 50% of the maximum stress. Under each set of CCDFs are their respective scaling collapses and their fits onto the predicted scaling function from Equation (2) of the main text. As lower stresses are used, finite-size effects in the slip statistics are reduced, removing the bump in the collapse function, and improving the collapse as shown in the figure. As shown above, looking at the collapses from left to right, the collapse shape deviates less from the scaling function, just as predicted. The remaining slight deviation from the scaling function of Figure 2 stems mainly from the fact that the packing fraction during the granular experiments is below random closed packed (at around 90% of random closed packed).*

**III. Prediction for probability density functions (PDF) or histograms of the slip sizes:**

The model predicts that histograms or PDFs of the slip-sizes at stress *F* scale as *D*(*S,F*) *~ S ̶ κ D_S_*(*S/S_max_*) with *κ = 1.5*. Here *S_max_* is the same stress-dependent cutoff size that is used in the main text and *D_S_*(*S/S_max_*) *~ exp*( ̶ *S/S_max_*) is an exponentially-decreasing cutoff function – i.e. for sizes *S<S_max_* the distribution *D*(*S,F*) follows power-law *S^-κ^*, while for *S > S_max_,* it drops off exponentially with *S*. For the analysis of experiments or observations with few events it can be advantageous to use complementary cumulative distribution functions (CCDFs) instead of binned histograms [*1*], as done in the main text. A main advantage of using CCDFs is that for CCDFs no binning is necessary.

**IV. Error Bar Analysis:**

For the error bars on our histograms a Bayesian technique was used with 95% confidence intervals. The method is explained in detail in [*38,39*].

**V. Details on how the parameters *kx, ky,* and *F_max_* are determined:**

1. **Procedure to determine *F_max_*:**
2. Take a set of data, and extract the stress at the beginning of every slip avalanche.

1. The highest stress value from (1) would be *F_max_* for that specific data set.
2. We then create a complementary cumulative distribution function (CCDF) of the sizes of the events whose stress at the beginning was between 50-85% of the F_max_. (Going closer to *F_max_* creates distortions in the CCDFs due to finite sample size effects).
3. Do steps 1-3 for all data sets. The resulting CCDFs are representative of the CCDFs near *F_max_*. Since the model predicts that the CCDFs near *F_max_* should have similar scaling behavior, we can test if the data fits this prediction. Figure 2 confirms that this prediction holds.
4. **Procedure to determine *k_x_* and *k_y_*:**

These constants are used to simplify the comparison between the statistics of events that span many magnitude scales.

1. We set *k_x_* and *k_y_* to be 1 and 1.56 for the Granular(Fore) data. This initial multiplication is arbitrary.
2. We then scale each of the rest of the CDFs by different values (given in Table 2 in the manuscript) until the CCDF is near the Granular(Fore) CCDF obtained in step (1). Note that multiplication of the CCDF with a constant only leads to a shift of the CCDF on a log log plot, it does not change the slope of the CCDF on the log log plot. Since we only wanted to compare the slopes of the curves, which give the exponent κ, this multiplication of the *x* and *y* axes is permissible.
3. Now the comparison is easy. Note that *k_x_ a*nd *k_y_* have NO PHYSICAL MEANING in our model, but are rather coefficients that translate the CCDFs onto each other for ease of comparison.
